# Detection capability of the Medical Imaging Projection System for sentinel lymph node biopsy in patients with breast cancer with and without neoadjuvant chemotherapy: a retrospective study

**DOI:** 10.1007/s12282-025-01712-8

**Published:** 2025-05-10

**Authors:** Marin Taguchi, Masahiro Takada, He Jiaxi, Yukiko Fukui, Hanako Shimizu, Ayane Yamaguchi, Kosuke Kawaguchi, Masahiro Kawashima, Nobuko Kawaguchi-Sakita, Masakazu Toi

**Affiliations:** 1 Department of Breast Surgery, Kyoto University Hospital, 54 Shogoin-kawahara-cho, Sakyou-ku, Kyoto City, Kyoto 606-8507 Japan; 2 Department of Breast Surgery, Kansai Medical University, 2-3-1, Shinmachi, Hirakata, Osaka 573-1191 Japan; 3 Department of Breast Surgery, Mie University Graduate School of Medicine, 2-174 Edobashi, Tsu City, Mie 514-0001 Japan; 4 Department of Clinical Oncology, Kyoto University Hospital, 54 Shogoin-kawahara-cho, Sakyou-ku, Kyoto City, Kyoto 606-8507 Japan; 5 Tokyo Metropolitan Cancer and Infectious Disease Center Komagome Hospital, 3-18-22 Honkomagome, Bunkyo-ku, Tokyo, 113-8677 Japan; 6 Present Address: Department of Breast Surgery, Shiga General Hospital, 5-4-30, Moriyama, Moriyama City, Shiga, 524-8524, Japan

**Keywords:** Breast cancer, Indocyanine green fluorescence method, Neoadjuvant chemotherapy, Projection mapping, Sentinel lymph node biopsy

## Abstract

**Background:**

The Medical Imaging Projection System (MIPS) projects fluorescence ICG images on the surgical field. In this study, we aimed to assess sentinel lymph node (SLN) identification by the MIPS in patients with and without neoadjuvant chemotherapy (NAC) administration and compare the utility of the MIPS with the radioisotope (RI) method.

**Methods:**

We retrospectively reviewed medical records of patients with primary breast cancer who underwent SLN biopsy using the MIPS at Kyoto University Hospital between April 2020 and December 2024. The primary endpoint was the identification rate of SLNs. The secondary endpoints included the number of positive SLNs and SLNs detected per patient.

**Results:**

The analysis included 470 procedures (448 patients), of which 56 (11.9%) were conducted after NAC. The identification rate of SLNs by the MIPS was 99.6% (95% confidence interval [CI], 98.5–99.9) in all procedures and 98.2% (95% CI, 90.6–99.7) after NAC. The median number of SLNs identified per patient was 3 (range, 2–4) by the MIPS and 2 (range, 1–3) by the RI method (P < 0.001). No significant difference was observed in the number of SLNs between patients who received NAC and those who did not (3 vs 3, P = 0.84). Seventy-eight positive SLNs were excised, all of which were accurately identified by the MIPS.

**Conclusions:**

This study suggested that the identification rate of SLNs by the MIPS was high regardless of the presence or absence of preceding systemic chemotherapy.

## Introduction

Breast cancer is the most commonly diagnosed cancer in the world. There were approximately 2.3 million new cases of breast cancer in women in 2020 [1] and it is presumed that its incidence rate will remain high for decades [2]. Multidisciplinary treatment, such as systemic therapy and radiotherapy, has improved the prognosis of patients with breast cancer. For patients with early breast cancer, the axillary nodal status is one of the important prognostic factors to make treatment decisions [3]. While axillary dissection has been a standard technique for axillary staging, sentinel lymph node (SLN) biopsy has become the standard of care for patients with early breast cancer [4, 5]. The patients with negative-SLNs can avoid axillary dissection without compromising prognosis. The randomized controlled trials have shown that patients with limited number of metastatic nodes in the SLNs can also avoid axillary dissection [6–9]. Several studies have investigated the feasibility of SLN biopsy after neoadjuvant chemotherapy (NAC) for patients who initially presented with clinically positive axillary nodes. These studies showed that the false-negative rate was > 10% in intent-to-treat population, but it was suggested that use of dual tracer or removal of three or more SLNs may reduce the false-negative rate to an acceptable level [10, 11]. Targeted axillary dissection (TAD) is a technique for removing biopsy-proven positive axillary lymph nodes in addition to SLNs in patients who received NAC. TAD reportedly reduces the false-negative rate [12–14].

Conventionally, the radioisotope (RI) and blue dye methods have been used to identify SLNs [4, 5]. In 2005, SLN identification with indocyanine green (ICG) fluorescence method was first reported [15], and since then, ICG fluorescence imaging has shown a high identification rate in several clinical studies [16, 17]. The meta-analyses have shown that there was no significant difference in SLN identification between the ICG fluorescence and RI methods and the ICG fluorescence method was superior to the blue dye method in terms of the identification rate. In addition, the ICG fluorescence method identified more SLNs per patient compared to the RI or blue dye methods, suggesting that the ICG fluorescence method may reduce the false-negative rate in SLN identification after NAC [18, 19].

The Medical Imaging Projection System (MIPS) is an innovative near-infrared (NIR) fluorescence imaging system that projects ICG images directly onto the surgical field using projection mapping technology [20]. The MIPS-assisted ICG fluorescence method provides continuous real-time navigation for SLN biopsy, eliminating the need for surgeons to shift their visual focus from the surgical field. Previous studies have reported a high SLN identification rate with MIPS, comparable to the conventional ICG fluorescence and RI methods [20, 21]. However, prior evaluations of the clinical utility of SLN biopsy using MIPS were limited by small sample sizes and a low proportion of patients with positive SLNs, particularly in post-NAC settings.

In this study, we aimed to evaluate SLN identification using the MIPS both with and without NAC administration and to compare its utility with the RI method.

## Patients and methods

### Study design and patients

We retrospectively reviewed medical records of patients with primary breast cancer who underwent SLN biopsy using the MIPS at Kyoto University Hospital between April 2020 and December 2024. The eligibility criteria included histologically confirmed breast cancer and patients who underwent SLN biopsy using the MIPS. The patients with initially clinical node positive tumors who received NAC were also included. The exclusion criteria included patients with a previous history of axial surgery. We used the following methods when we performed SLN biopsies using MIPS and RI methods. The surgeon identified and excised the SLNs using MIPS, followed by the RI method to identify and excise any remaining SLNs. Each SLN removed was checked for fluorescence or RI signal and recorded. The study protocol was approved by the institutional review board of Kyoto University Hospital (approval no.: R4913).

We collected data on age, sex, body mass index (BMI), tumor histology, subtype, administration of NAC, combined use of the RI method, number of SLNs identified, operating time of SLN biopsy, and pathology reports. A detailed description of the MIPS and its surgical procedure have been reported previously [22]. MIPS is approved in Japan as a class II medical device, and in this study period, commercially available MPIS was used in clinical practice.

### Statistical analysis

The primary endpoint of this study was the SLN identification rate using the MIPS, defined as the proportion of patients in whom at least one SLN was detected with the system. The secondary endpoints included the number of positive SLNs, number of SLNs detected per patient using the MIPS, operating time for SLN biopsy, and number of patients with axillary recurrence after SLN biopsy.

The SLN identification rate and number of positive SLNs were analyzed for all procedures and specifically for those performed after NAC. The number of SLNs detected was compared between patient groups stratified by clinical factors, such as NAC administration or BMI, using the Wilcoxon rank-sum test. In cases where both the MIPS and RI methods were used, the number of SLNs identified and positive SLNs were compared between methods. The operating times were also compared between methods, with or without combination with the RI method, using the Wilcoxon rank-sum test.

Statistical significance was assessed with a two-sided level of 0.05. All analyses were conducted using the JMP® Pro software (version 17.0.0; SAS Institute, Inc., Cary, NC, USA).

## Results

A total of 452 patients (including 22 patients with bilateral breast cancer) underwent SLN biopsy using the MIPS between April 2020 and December 2024. Four patients with unilateral breast cancer had a prior history of axial surgery. The analysis was based on a total of 470 procedures (448 patients). The baseline characteristics of the patients and tumors are presented in Table 1. The median age was 59 (range, 25−87) years and the median BMI was 21.7 (range, 15.6−43) kg/m^2^. The procedures performed for ductal carcinomas in situ accounted for 18.5%, and approximately 80% of procedures were performed for T1–T2 tumors. There were eight patients (1.7%) with clinically node-positive breast cancer and six of them underwent NAC. Of the 470 procedures, 56 (11.9%) were conducted after NAC. Both the MIPS and RI methods were used in 327 (69.6%) procedures.

**Table 1:** Patient and tumor characteristics

Factors	N	%
All procedures	470	
Age (years)	Median (range)	59 (25–87)
BMI	Median (range)	21.7 (15.6–43.0)
Tumor stage		
Tis	87	18.5
T1 mi	5	1.1
T1a	8	1.7
T1b	59	12.6
T1c	172	36.6
T2	131	27.9
T3	6	1.3
T4	2	0.4
Nodal stage		
N0	462	98.3
N1	8	1.7
Histology		
DCIS	87	18.5
IDC	326	69.4
ILC	28	6.0
Others	29	6.2
Subtype		
HR +, HER2−	303	64.5
HR +, HER +	30	6.4
HR−, HER2 +	13	2.8
Triple negative	37	7.9
Unknown	87	18.5
Neoadjuvant chemotherapy		
Yes	56	11.9
No	414	88.1
Combined use of radioisotope method		
Yes	327	69.6
No	143	30.4
Operative procedure		
Bp	196	41.7
Bt	274	58.3
Axillary dissection		
Yes	30	6.4
No	440	93.6

BMI, body mass index; DCIS, ductal carcinomas in situ; IDC, invasive ductal carcinoma; ILC, invasive lobular carcinoma

The identification rate of SLNs by the MIPS was 99.6% (468/470: 95% confidence interval [CI], 98.5–99.9) in all procedures and 98.2% (55/56: 95% CI, 90.6–99.7) in procedures after NAC.

Of 1480 SLNs excised among the patients who underwent SLN biopsy using by the MIPS, there were 114 positive SLNs. Of 180 SLNs excised among patients who have undergone NAC, four positive SLNs were detected.

The median number of SLNs identified per patient was 3 (range, 2–4) by the MIPS and 2 (range, 1–3) by the RI method (P < 0.001) (Table 2). The median number of SLNs identified by the MIPS per patient who had received NAC was 3 (range, 2–4). This study showed no significant difference in the number of SLNs between patients who received NAC and those who did not (3 vs 3, P = 0.84). There was no significant difference in the median number of SLNs detected by the MIPS between patients with a high BMI (≥ 22 kg/m^2^) and those with a low BMI (< 22 kg/m^2^) (3 vs 3, *P* = 0.41).

**Table 2:** Number of SLNs identified by the MIPS

Characteristics		N	Median	IQR	P-value
BMI	< 22	244	3	2–4	0.41
	≥ 22	226	3	2–4	
NAC	No	414	3	2–4	0.84
	Yes	56	3	2–4	
Breast surgery	Bp	196	3	2–4	0.68
	Bt	274	3	2–4	

BMI, body mass index; NAC, neo adjuvant chemotherapy; IQR, interquartile range

A total of 1062 SLNs were excised in 327 procedures, in which the MIPS and RI methods were used (Table 3a). Among them, 657 (62%) SLNs were identified by both the MIPS and RI methods, 394 (37%) only by the MIPS, four (0.38%) only by the RI method, and seven (0.66%) by neither the MIPS nor RI method (palpation). A total of 78 positive SLNs were excised and all of them were identified by the MIPS. No positive SLNs were identified by the RI method alone. Among procedures which were conducted after NAC, 147 SLNs were identified in 44 procedures, in which both the MIPS and RI methods were used (Table 3b). All SLNs were identified by the MIPS. There were four positive SLNs, which were identified by both the MIPS and RI methods.

**Table 3:** Number of SLNs among cases using MIPS and RI

(a) Among all procedures (n = 327):		
SLN biopsy procedures	Identified SLNs (total N = 1062)	Positive SLNs (total N = 78)
MIPS and RI	657	56
MIPS only	394	22
RI only	4	0
Neither MIPS nor RI	7	0

(b) Among procedures following NAC (n = 44):		
SLN biopsy procedures	Identified SLNs (total N = 147)	Positive SLNs (total N = 4)
MIPS and RI	97	4
MIPS only	50	0
RI only	0	0
Neither MIPS nor RI	0	0

MIPS, medical imaging projection system; NAC, neoadjuvant chemotherapy; RI, radioisotope; SLN, sentinel lymph node

The median operating time for SLN biopsy using only the MIPS was 26 (range, 19–39) min and using the MIPS and the RI method was 31 (range, 22–45) min (*p* < 0.01) (Table 4). At a median follow-up of 32 (range, 2–58) months, no patient experienced axillary recurrence following SLN biopsy.

**Table 4:** Operating time using MIPS according to the combination of the RI method

Method	N	Median	IQR	P-value
MIPS and RI	326	31	22–45	< 0.01
MIPS only	142	26	19–39	

IQR, interquartile range; MIPS, medical imaging projection system; RI, radioisotope

## Discussion

This study reinforces our previous findings demonstrating a high SLN identification rate using the ICG fluorescence method with MIPS [20, 21]. Notably, even among patients who received NAC, the SLN identification rate with MIPS remained high. There was no significant difference in the median number of SLNs detected per patient by MIPS between those who received NAC and those who did not. In procedures utilizing both the MIPS and RI methods, all positive SLNs were identified by MIPS, with none identified solely by the RI method.

A prior meta-analysis reported SLN identification rates of 88.6–100% with the ICG fluorescence method and 85–100% with the RI method [23]. A recent meta-analysis focusing on post-NAC SLN biopsies showed an identification rate of 90.6% (95% CI, 89.1–92.2) [24]. In this study, the SLN identification rate by MIPS was 99.6% (95% CI, 98.5–99.9) across all patients and 98.2% (95% CI, 90.6–99.7) in those who received NAC. These findings indicate that the SLN identification rate with MIPS is comparable to conventional methods, regardless of NAC status. The near-infrared camera equipped in MIPS is the same as the conventional one. Therefore, MIPS showed a high SLN identification rate similar to the conventional ICG fluorescence methods. Compared to conventional near-infrared camera systems, MIPS has the advantage of real-time visibility of the fluorescence signal in the operative field, allowing for a smoother surgical procedure.

Accurate SLN biopsy is critical for early-stage breast cancer management, as the number of positive SLNs informs decisions regarding additional local axillary treatment and systemic adjuvant therapy. In this study, all positive SLNs excised were identified by MIPS, with none detected by the RI method alone. These results align with previous research [21], suggesting that the RI method may not provide additional value over MIPS in identifying positive SLNs. The results of the meta-analysis indicate that the ICG fluorescence method has a similar SLN identification rate as the RI method [18, 19]. Sugie et al. also showed that the ICG fluorescence and RI combination had a significantly higher SLN identification rate than RI alone [17]. Because this study was a retrospective study, it was not possible to conclude whether the RI method should be used in addition to MIPS, and further studies are needed.

The SLN identification rate is reportedly lower and the false-negative rate is higher in patients who received NAC compared to those who did not [11, 25]. A recent meta-analysis indicated that removing at least three SLNs may reduce the false-negative rate in patients post-NAC [24]. In our study, the median number of SLNs identified per patient using the MIPS was 3 (95% CI, 2–4), consistent even among patients post-NAC, where 3 (range, 2–4) SLNs were identified. As previous studies have shown, the use of dual tracers for SLN detection may further reduce the false-negative rate [10, 11, 24].

Our study included eight patients with clinically node-positive breast cancer and six of them underwent NAC. All procedures were conducted both by the MIPS and the RI method, and all SLNs including metastatic SLNs excised were identified by the MIPS. Among six procedures conducted after NAC, there were no metastatic nodes in five procedures leading to avoiding axillary dissection. Although TAD was not routinely conducted in our facility, considering that there was no patient who experienced axillary recurrence following SLN biopsy, use of dual tracer and detecting stable number of SLNs could improve the feasibility of SLN biopsy.

Our study found significant difference in SLN biopsy operating time between procedures conducted solely with MIPS and those combining MIPS and RI methods. Although the addition of the RI method may lead to longer operating time than only by the MIPS, it was suggested that the use of dual tracer may reduce the false-negative rate [10, 11].

The MIPS-assisted ICG fluorescence method enables real-time navigation during SLN biopsy, potentially streamlining the procedure. In this study, the median operating time for SLN biopsy was 26 (range, 19–39) min using MIPS alone and 31 (range, 22–45) min with MIPS and RI methods combined. These durations are comparable to SLN biopsy times using conventional methods [26, 27]. The operating times can be influenced by various factors, such as surgeon skill level or patient BMI. Although we did not collect specific data on SLN biopsy times with conventional NIR devices, these findings suggest that MIPS does not prolong surgery compared to conventional methods.

A systematic review reported an axillary recurrence rate of 0.3% in clinically node-negative patients with breast cancer and SLN-negative findings [28]. For patients who initially presented with clinically positive axillary nodes but became node-negative after NAC, the reported axillary recurrence rate was < 1% [29, 30]. Consistent with these findings, no axillary recurrences have been observed in our cohort following SLN biopsy to date.

The current study had some limitations. First, as this study was conducted at a single center, the generalizability of the findings may have been limited. Further multi-institutional studies are necessary to validate the efficacy and utility of MIPS. Second, procedures conducted after NAC accounted for only 12% of the total cohort, and the sample size of patients with clinical node-positive breast cancer was small. Although the results demonstrated high SLN identification rates with MIPS in patients who have undergone NAC, larger studies are required to confirm these findings and establish broader applicability. Third, the follow-up duration at the time of this investigation was insufficient to thoroughly evaluate the recurrence rate. Additionally, safety assessments of the procedures were not included in this study. Extended follow-up and more comprehensive analyses are needed to address these gaps.

Despite these limitations, MIPS achieved an SLN identification rate of 99.6% and 98.2% overall and in patients who received NAC, respectively, showcasing its robustness across varied clinical scenarios. The MIPS method can serve as a reliable approach to identifying positive SLNs and consistently removing a stable number of SLNs, regardless of whether systemic chemotherapy was administered. Furthermore, it holds potential to reduce the false-negative rate, supporting its utility in SLN biopsies.

## Data Availability

The data that support the findings of this study are available from the corresponding author upon reasonable request.
